# Dissecting the effect of workplace exposures on workers’ rating of psychological health and safety

**DOI:** 10.1002/ajim.22964

**Published:** 2019-03-27

**Authors:** Avinash Ramkissoon, Peter Smith, John Oudyk

**Affiliations:** ^1^ Epidemiology Division Dalla Lana School of Public Health Toronto Ontario; ^2^ Institute for Work & Health Toronto Ontario; ^3^ Department of Epidemiology and Preventive Medicine Monash University Melbourne Australia; ^4^ Occupational Health Clinics for Ontario Workers Toronto Ontario

**Keywords:** Canada, employment, factor analysis, mental health, surveys and questionnaires

## Abstract

**Objectives:**

To validate the factor structure of the Copenhagen Psychosocial Questionnaire (COPSOQ) in a North American population and dissect the associations between psychosocial factors and workplace psychological health and safety.

**Methods:**

Confirmatory factor analysis and multivariate linear regression were used to determine the associations between COPSOQ dimensions and a global rating of workplace psychological health and safety. Models were stratified by sex, gender roles, and age.

**Results:**

The COPSOQ factor structure was verified among Canadian workers. Three factors were found to significantly contribute to the global rating of the psychological health and safety for all workers. Few differences were observed across sex, gender roles, and age.

**Conclusions:**

This study identified dimensions of the psychosocial work environment that are strongly associated with the global rating of workplace psychological health and safety. Using a standardized questionnaire like the COPSOQ allows for comparisons over time, between different industries, and worker populations.

## 1 INTRODUCTION

Psychological health and safety in the workplace is a topic of increasing interest in most developed economies. This interest is due, in part, to increased recognition of the role that work conditions play in the development of mental health conditions, and a shift away from a primarily goods‐producing labor market.^1^, ^2^ For example, in 2013, the Canadian Standards Association released a voluntary standard related to psychological health and safety in the workplace.^3^ The incorporation of psychological health and safety within the broader context of health and safety at work recognizes that there has been a reduction of visible, quantifiable hazards, which have acute effects on health, and a concurrent emergence of concerns over less visible, difficult to measure hazards in the workplace.^4^ In addition, poor psychosocial health and safety has been associated with negative health outcomes among workers, as well as impacts on workplace productivity through reduced employee engagement, and less shared problem solving.^5^, ^6^

Likely key predictors of psychological health and safety are dimensions of the psychosocial work environment.^7^, ^8^ The psychosocial work environment has been defined as the sociostructural opportunities available in the workplace that allow individuals have their expectations met with regard to well‐being, productivity, opportunities for learning, and positive interactions with others.^8^ Many commonly used measures to assess the psychosocial work environment, such as the demand‐control model and the effort‐reward imbalance model, were developed more than two decades ago.^9^, ^10^ As such, it is not clear if these measures still capture all aspects of the psychosocial work environment that are relevant and important to workers.^3^ The Copenhagen Psychosocial Questionnaire (COPSOQ) is one of the more recently developed measures of the psychosocial work environment, capturing a broad range of psychosocial dimensions.^7^ While this measure has been validated in a variety of countries and languages, it has not been validated in a North American context, nor in any English‐speaking population.^11^, ^12^ Although the COPSOQ captures a wide number of dimensions, it is not clear whether some of these dimensions are more important than others in relation to perceptions of psychological health and safety.^3^ As such, the objective of this study was to assess the validity of the COPSOQ's factor structure in a North American context, and to examine the relationship between psychosocial dimensions of the COPSOQ and perceptions of psychological health and safety at work.

Some studies have documented that the relationship between psychosocial work exposures and mental and physical health outcomes differs for men and women.^13^, ^14^, ^15^, ^16^ As such, there is also the potential that the relationship between psychosocial work exposures and the psychological health and safety might differ for men and women, or for those with more masculine or feminine roles in relation to the labor market. Feminine labor market roles include having a greater responsibility for child care or other household responsibilities, working fewer hours relative to one's partner, and working in female‐dominated occupational groups, whereas masculine labor market characteristics include being the sole wage earner and working in male‐dominated occupational groups.^17^ In addition, from a life course perspective, the relationships between psychosocial exposures and psychological health and safety might also differ across age groups.^18^ Consequently, the secondary objective of this study is to examine the extent to which the relationship between psychosocial work exposures and psychological health and safety differ for men and women, for those with masculine and feminine labor market roles, and across age groups.

## 2 METHODS

### 2.1 Data and sampling

We used data from a sample of 4113 labor market participants across Canada, collected between February and March 2016. Recruitment was conducted by a professional survey company, and participants were drawn from a representative panel of approximately 90 000 Canadians who have agreed to participate in surveys “from time to time”. To be eligible to complete the survey, respondents had to be currently working in an organization with six or more employees. All surveys were completed through an on‐line survey platform that did not allow respondents to complete the survey more than once, and was available in either English or French. No personal identifying information was collected on any of the respondents as part of the survey. The questionnaire used for this study is available in the Supporting Information Material. A total of 56 257 respondents were invited to complete the survey, of which 5697 agreed to participate (10% response rate). Of this sample, 1584 did not meet the eligibility criteria, leaving a sample of 4113 respondents who completed the survey. The demographic characteristics of the study population were compared with the broader Canadian labor market using the Labor Force Survey (2016), available from Statistics Canada. This information is available in the Supporting Information Material for this study. Approval for this study was received from the University of Toronto's Health Sciences Research Ethics Board.

### 2.2 Main dependent variable: psychological health and safety

The global rating of workplace psychological health and safety was measured using a single question “How would you rate the psychological health and safety climate in your workplace?” with response options being healthy/supportive, good, fair, neutral, not so good, poor, and toxic. The use of this outcome measure has not been previously validated; however, in the absence of another measure of psychological health and safety, this question provides an initial assessment of perception of psychological health and safety among workers. As the reliability of this single‐item question is not currently established, we conducted a test‐retest analysis of this question in subsample of 91 respondents, who indicated their jobs had not changed in between the initial survey and the retest. The median time between the initial survey and the follow‐up survey was 18 days. This analysis indicated good‐to‐excellent reliability for this single item, with an inter‐class correlation of ICC(2,1) = 0.804.^19^, ^20^

### 2.3 Main independent variable: psychosocial work exposures

Psychosocial work exposures were assessed using the COPSOQ. The COPSOQ I and II were developed by the Psychosocial Department at the National Institute of Occupational Health in Denmark.^7^, ^11^ The COPSOQ International Network is now responsible for updating and publishing new versions of the questionnaire. The benefit of using the COPSOQ, as opposed to other questionnaires, is that the standardized nature of the questionnaire allows for a valid comparison of results between worker populations and subpopulations, between workplaces, between industries, and between countries.^7^ The survey used in this study had 35 questions associated with 19 psychosocial dimensions, which were drawn from the COPSOQ II (short) and a beta version of the COPSOQ III (core). The following dimensions were captured through the survey, with the number of items informing them in brackets: quantitative demands (3), work pace (2), emotional demands (3), influence at work (2), possibilities for development (3), meaning of work (2), commitment to the workplace (2), predictability (2), rewards (2), role clarity (2), role conflict (3), quality of leadership (3), social support from supervisors (2), social support from colleagues (1), social community at work (1), job insecurity (3), work‐life conflict (3), vertical trust (2), and organizational justice (2). All questions were coded such that higher values were associated with a more negative exposure.

### 2.4 Covariates

Variables that were considered as confounders for the relationship between psychosocial exposures and the global rating of the psychological health and safety were collected as part of the survey, and included province or territory of employment, industry of employment, language of survey response, workplace size, level of education, shift schedule, employment in a management position, and working at more than one job.

### 2.5 Effect modifiers

Effect modifiers included the sex reported, age group, and gender roles, which were also captured through the survey. The sex question included an option for “transgendered”, but too few respondents endorsed this category to enable analysis for this group; as such it was dichotomized to men and women. Age was grouped as under 30 years of age, 30 to 50 years of age, and more than 50 years of age. For gendered labor market roles (masculine/intermediate/feminine), we used a series of four questions relating to primary earner status, hours per week spent on housework, primary responsibility for doing housework, and primary responsibility for caring for those who need care. From responses to these questions, we estimated a scale which ranged from 0 (most masculine labor market role) to 13 (most feminine labor market role). This approach to defining gender is consistent with previous studies.^17^, ^21^, ^22^ The scale was divided into quartiles; the first quartile corresponding to masculine gender roles, the fourth quartile corresponding to feminine gender roles, and the second and third quartiles corresponding to intermediate gender roles.

### 2.6 Analysis

Initial descriptive analyses examined the distribution of all study variables. Confirmatory factor analysis (CFA) was then used to validate the factor structure of the COPSOQ. The original theoretical model contained 35 items, influenced by 19 latent factors, our COPSOQ dimensions. For this analysis, two dimensions that are informed by single items in the COPSOQ (social support from colleagues and social community at work) were not examined. A linear model was constructed in the following format for each item: $V_{1}=L_{I_{1}\text{:}F_{1}}F_{1}+E_{V_{1}}$, where *I* is the item, *F* is the factor, *L* is the loading for the specified pathway, and *E* is the error term. The variance of each factor was set to one, and covariance between all factors was allowed. Covariance between error terms for each item were allowed within latent constructs to improve model fit. The model fit parameters were estimated, including standardized root mean square residual, goodness‐of‐fit index (GFI), Bentler comparative fit index (CFI), and root mean square error of approximation (RMSEA). Interfactor correlations, and factor loadings for each item were also estimated.

Linear regression models were then used to estimate the association between each psychosocial work exposure and psychological health and safety, accounting for potential confounders. Pairs of factors with strong correlations (>0.85, indicating >70% shared variance) were merged into a single factor under the assumption that they are measuring the same (or a very similar) underlying construct, and to reduce multicollinearity in our regression analyses. A subsequent model included all psychosocial exposures in a single model. To enable comparability across exposures (given the differing number of items that each exposure was constructed from) the scores for each measure were rescaled to a 0 to 10 scale, where 0 is the best possible score on the dimension and 10 is the worst possible score on the dimension.

To examine potential effect modification by sex, age, and gendered labor roles, we ran a series of stratified regression models to examine potential differences in the relationship between psychosocial exposures and psychological health for men and women, across gendered labor market roles, and across age groups. To examine differences across subgroups we compared coefficients and standard errors from stratified models to examine if they were statistically different from each other.^23^ The results from this post hoc analysis are almost identical to results that would be obtained by specifying interactions between the modifying variable and each psychosocial work factor, and other covariates, in a single regression model. All analyses were conducted with SAS (Cary, NC) version 9.3 TS Level 1M2 for Windows.

### 2.7 Institution and ethical approval and informed consent

Ethical approval was obtained from the Health Sciences Research Board at the University of Toronto. Written informed consent was obtained from study participants.

## 3 RESULTS

Of the initial sample of 4113 respondents, 24 (<1%) did not respond to the global rating of the psychological health and safety climate question. Another 435 (11%) of respondents were missing information to one or more items on psychosocial work exposures. For 401 of these respondents, we were able to impute values for the missing item based on responses to other items within the same psychosocial dimension. Another 85 (2%) respondents were missing information on the covariates and an additional 52 (1%) were missing information on one of the effect modifiers; for these 52 individuals, the missing responses were for items used to construct the gender role index. After removing these respondents, this left an analytical sample of 3919 respondents (95% of the initial sample). The sample recruited for this study was on average older, from larger organizations, and more likely to be used in educational services and other service industries compared with the used Canadian labor force during the same time period. However, participants in this study were from a wide variety of industries and workplace sizes.

Validation of the factor structure via CFA yielded the hypothesized 17‐factor solution. This model showed excellent model fit, as assessed by various fit indices, RMSEA = 0.044 (90% confidence interval [CI]: 0.043, 0.046), GFI = 0.95, and CFI = 0.95. A correlation matrix was generated for the 17 factors (Table 1). There were three pairs of factors with correlations greater than 0.85: meaning of work and commitment to the workplace (0.851), predictability and rewards (0.858), and vertical trust and organizational justice (0.937). To ensure a lack of multicollinearity and parsimony for the regression models, these highly correlated factors were combined into single dimensions, and then rescored to a 0 to 10 scale.

**Table 1:** Correlation between factor scores for dimensions of the COPSOQ. Employed Canadians working in workplaces with more than five employees (*N* = 3919)

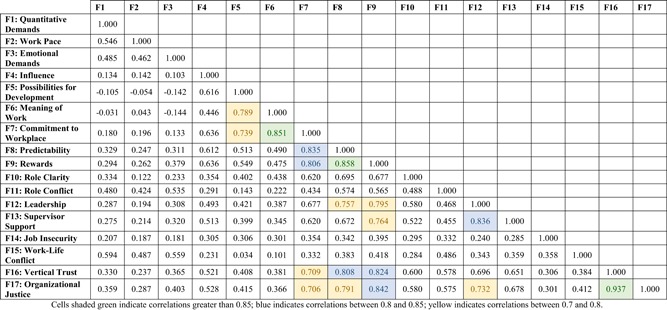

Table 2 presents the distribution of the main outcome and psychosocial work exposures for men and women in our analytical sample of 3919 respondents. In addition, at the bottom of the table is the distribution of gender roles in relation to work for men and women. The distribution of responses for the single‐item question on psychological health and safety showed good distribution, with no noticeable ceiling or floor effects. Similarly, scores across all psychosocial measures show variation in the study sample. Differences between the sexes were noted in responses to the questions on the psychological health and safety, with more men rating their psychological work environment as healthy and more women rating their psychological work environment less favorably. Differences between males and females were also observed across psychosocial work exposures; compared with men, women reported a more negative work pace, higher emotional demands, and lower influence at work. However, women also reported a more positive meaning of work and commitment to the workplace, role clarity, role conflict, and lower job insecurity. As expected, women were more likely to have feminine gender roles in relation to work, while men had more masculine roles; however, 13% of women had masculine gender roles and 15% of men had feminine gender roles.

**Table 2:** Demographic characteristics of study participants, stratified by sex

	Males (*N* = 2026)	Females (*N* = 1893)	*P* value for difference between men and women
Outcome: workplace psychological health and safety climate	Frequency (%)	Frequency (%)	
Healthy/supportive	347 (17%)	258 (14%)	<0.01
Good	600 (30%)	588 (31%)	
Fair	436 (22%)	399 (21%)	
Neutral	164 (8%)	136 (7%)	
Not so good	274 (14%)	323 (17%)	
Poor	107 (5%)	95 (5%)	
Toxic	98 (5%)	94 (5%)	
Psychosocial exposures			
Quantitative demands	4.29 (2.13)	4.38 (2.25)	0.21
Work pace	5.85 (2.26)	6.15 (2.32)	<0.01
Emotional demands	4.47 (2.52)	5.07 (2.52)	<0.01
Influence at work	5.02 (2.58)	5.64 (2.47)	<0.01
Possibilities for development	3.11 (2.20)	3.12 (2.12)	0.89
Meaning of work + commitment to the workplace	3.55 (2.44)	3.35 (2.33)	0.01
Predictability + rewards	4.40 (2.49)	4.45 (2.41)	0.47
Role clarity	3.16 (2.40)	2.92 (2.26)	<0.01
Role conflict	4.71 (2.44)	4.52 (2.50)	0.01
Quality of leadership + social support from supervisors	4.22 (2.55)	4.19 (2.62)	0.78
Social support from colleagues	2.94 (2.45)	2.79 (2.41)	0.04
Social community at work	2.22 (2.17)	2.27 (2.07)	0.47
Job insecurity	3.46 (2.64)	3.20 (2.48)	<0.01
Work life conflict	5.89 (2.20)	5.85 (2.24)	0.54
Vertical trust + organizational justice	4.03 (2.41)	3.97 (2.35)	0.44
Gender role in relation to work			
Masculine (lowest quartile)	758 (37%)	253 (13%)	
Intermediate	957 (47%)	791 (42%)	<0.01
Feminine (highest quartile)	311 (15%)	849 (45%)	

Employed Canadians working in workplaces with more than five employees (*N* = 3919). Psychosocial exposures were adjusted to a 0 to 10 scale, with higher scores indicating a more negative exposure. The outcome was scored such that a worse workplace psychological health and safety climate was given a higher score on a scale of 1 to 7.

A *P* < 0.05 indicates a significant difference between the male and female groups at the 95% confidence level.

Table 3 presents the results of the linear regression models examining the relationship between psychosocial work exposures and perceived psychological health and safety at work. Model 1 includes each psychosocial work exposure individually, and all covariates. Model 2 includes all psychosocial work exposures in the same model, and all covariates. After adjusting for only study covariates, all psychosocial work exposures were related to psychological health and safety, with more negative exposures associated with a worse workplace psychological health and safety. Based on the standardized β coefficients from the model, the strongest associations were observed for quality of leadership and social support from supervisors (β = 0.388), predictability and rewards (β = 0.457), and vertical trust and organizational justice (β = 0.486), which are the three strongly correlated pairs of dimensions. The weakest associations were observed for work pace (β = 0.186), job insecurity (β = 0.192), social support from colleagues (β = 0.219), and quantitative demands (β = 0.230).

**Table 3:** Standardized ordinary least‐squared linear regression estimates for dimensions of the COPSOQ and perceived workplace psychological health and safety climate in the workplace

	Model 1			Model 2		
COPSOQ dimensions	β Coefficient	SE	*P* value	β Coefficient	SE	*P* value
Quantitative demands	0.230	0.012	<0.001	0.017	0.010	0.09
Work pace	0.186	0.011	<0.001	0.010	0.009	0.27
Emotional demands	0.289	0.010	<0.001	0.093	0.009	**<0.001**
Influence at work	0.281	0.010	<0.001	0.052	0.009	**<0.001**
Possibilities for development	0.236	0.013	<0.001	−0.028	0.012	**0.02**
Meaning of work + commitment to the workplace	0.341	0.010	<0.001	0.069	0.012	**<0.001**
Predictability + rewards	0.457	0.008	<0.001	0.118	0.015	**<0.001**
Role clarity	0.306	0.010	<0.001	−0.034	0.010	**0.001**
Role conflict	0.323	0.010	<0.001	0.017	0.010	0.08
Quality of leadership + social support from supervisors	0.388	0.008	<0.001	0.076	0.011	**<0.001**
Social support from colleagues	0.219	0.010	<0.001	−0.015	0.009	0.11
Social community at work	0.312	0.012	<0.001	0.048	0.011	**<0.001**
Job insecurity	0.192	0.010	<0.001	0.019	0.008	**0.02**
Work life conflict	0.229	0.009	<0.001	0.028	0.008	**<0.001**
Vertical trust + organizational justice	0.486	0.008	<0.001	0.227	0.013	**<0.001**

Employed Canadians working in workplaces with more than five employees (*N* = 3919). Model 1 was adjusted for age, sex, gender role, language of interview, province, industry, education, shift schedule, workplace size, if working in a managerial occupation, multiple job‐holding, and full‐time employment. Model 2 was additionally adjusted for all other psychosocial exposures. Psychosocial exposures were adjusted to a 0 to 10 scale, with higher scores indicating a more negative exposure. The outcome was measured using a single question “How would you rate the workplace psychological health and safety climate in your workplace?” with response options being healthy/supportive, good, fair, neutral, not so good, poor, and toxic. The outcome was scored such that a worse workplace psychological health and safety climate was given a higher score on a scale of 1 to 7. Positive β coefficients indicate a higher level of the psychosocial exposure is associated with a worse psychological health and safety climate. Bold *P* values indicate a significant difference between compared β coefficients at the 95% confidence level.

Abbreviation: COPSOQ, Copenhagen Psychosocial Questionnaire.

The inclusion of all psychosocial work exposures in Model 2 led to attenuation of effects, given the moderate‐to‐strong correlations observed between several dimensions. The strongest associations were observed for vertical trust and organizational justice, predictability and rewards, and influence at work. No statistically significant association was observed for quantitative demands, work pace, role conflict, and social support from colleagues, and an inverse association was observed between possibilities for development and workplace psychological health and safety.

Stratified models for men and women, gender roles, and age groups are presented in Tables 4, 5, 6. Limited differences in associations were observed across subgroups, with one statistically significant difference in estimates observed for men and women, two across gender role groups, and three across age groups. There was a stronger association between work pace and workplace psychological health and safety among women compared with men (*P* = 0.04). Across gender roles, there were differences with respect to emotional demands and social support from colleagues. Among people reporting masculine work roles, emotional demands had a stronger association with global psychological safety at work compared with people reporting feminine work roles (*P* = 0.02). People reporting masculine work roles also had a stronger, inverse association between social support from colleagues and the global psychosocial health and safety, compared with intermediate gender roles (*P* = 0.03). A stronger association was observed between quantitative demands and a worse psychological health and safety rating among respondents 30 to 50 years of age, compared with both younger and older respondents (*P* = 0.03, *P* = 0.05). Lastly, possibilities for development had a stronger inverse association among 30 to 50‐year‐old respondents, compared with older respondents (*P* = 0.03). Across all subgroups, greater vertical trust and organizational justice, and quality of leadership and social support from supervisors were significantly associated with a better global rating of psychological health and safety. More positive scores on meaning of work and commitment to the workplace, and predictability and rewards were associated with a better global rating of workplace psychological health and safety in all subgroups but one.

**Table 4:** Standardized ordinary least‐squared linear regression estimates for dimensions of the COPSOQ and perceived psychological health and safety in the workplace, stratified by sex

	Males (*N* = 2026)		Females (*N* = 1893)		
COPSOQ dimensions	β Coefficient	SE	β Coefficient	SE	P value for M vs F
Quantitative demands	0.026	0.014	0.009	0.015	0.42
Work pace	−0.009	0.013	**0.030**	**0.013**	**0.04**
Emotional demands	**0.099**	**0.013**	**0.086**	**0.014**	0.51
Influence at work	**0.044**	**0.012**	**0.059**	**0.013**	0.39
Possibilities for development	**−0.034**	**0.016**	−0.024	0.017	0.66
Meaning of work + commitment to the workplace	**0.071**	**0.016**	**0.074**	**0.017**	0.92
Predictability + rewards	**0.101**	**0.020**	**0.135**	**0.022**	0.25
Role clarity	−0.018	0.014	**−0.056**	**0.015**	0.07
Role conflict	0.008	0.014	0.027	0.014	0.35
Quality of leadership + social support from supervisors	**0.086**	**0.015**	**0.061**	**0.016**	0.27
Social support from colleagues	−0.012	0.013	−0.020	0.014	0.70
Social community at work	0.027	0.015	**0.069**	**0.017**	0.06
Job insecurity	0.015	0.011	0.021	0.011	0.68
Work life conflict	**0.036**	**0.011**	0.020	0.011	0.31
Vertical trust + organizational justice	**0.245**	**0.018**	**0.213**	**0.018**	0.22

Employed Canadians working in workplaces with more than five employees (*N* = 3919). Adjusted for age, gender role, language, workplace size, province or territory of employment, industry of employment, level of education, shift schedule, management role, and multiple employment. Psychosocial exposures were adjusted to a 0 to 10 scale, with higher scores indicating a more negative exposure. The outcome was measured using a single question “How would you rate the workplace psychological health and safety climate in your workplace?” with response options being healthy/supportive, good, fair, neutral, not so good, poor, and toxic. The outcome was scored such that a worse workplace psychological health and safety climate was given a higher score on a scale of 1 to 7. Positive β coefficients indicate a higher level of the psychosocial exposure is associated with a worse psychological health and safety climate. Bold β coefficients and SE indicate a significant difference from the null at the 95% confidence level. Bold *P* values indicate a significant difference between compared β coefficients at the 95% confidence level. *N*_Male_ = 2026, model *R* ^2^_Male_ = 0.60; *N*_Female_ = 1893, model *R* ^2^_Female_ = 0.58.

Abbreviation: COPSOQ, Copenhagen Psychosocial Questionnaire.

**Table 5:** Standardized ordinary least‐squared linear regression estimates for dimensions of the COPSOQ and perceived psychological health and safety in the workplace, stratified by categories of gendered labor market roles

	Masculine (N = 1011)		Intermediate (N = 1748)		Feminine (N = 1160)		*P* values for difference		
COPSOQ dimensions	β Coefficient	SE	β Coefficient	SE	β Coefficient	SE	M vs F	M vs I	I vs F
Quantitative demands	0.021	0.020	0.010	0.016	0.015	0.018	0.83	0.68	0.85
Work pace	−0.007	0.019	0.006	0.015	**0.037**	**0.016**	0.07	0.58	0.14
Emotional demands	**0.126**	**0.018**	**0.090**	**0.015**	**0.066**	**0.017**	**0.02**	0.13	0.31
Influence at work	**0.058**	**0.017**	**0.059**	**0.014**	**0.041**	**0.016**	0.47	0.94	0.37
Possibilities for development	−0.039	0.023	−0.036	0.018	−0.016	0.021	0.45	0.91	0.47
Meaning of work + commitment to the Workplace	0.033	0.023	**0.085**	**0.018**	**0.074**	**0.021**	0.19	0.07	0.68
Predictability + rewards	**0.114**	**0.029**	**0.097**	**0.023**	**0.151**	**0.027**	0.34	0.65	0.13
Role clarity	−0.012	0.020	−0.028	0.016	**−0.062**	**0.019**	0.07	0.53	0.16
Role conflict	−0.007	0.019	**0.032**	**0.015**	0.008	0.017	0.57	0.12	0.30
Quality of leadership + social support from supervisors	**0.075**	**0.021**	**0.087**	**0.017**	**0.056**	**0.021**	0.52	0.67	0.25
Social support from colleagues	**−0.051**	**0.019**	−0.001	0.014	−0.010	0.018	0.11	**0.03**	0.68
Social community at work	0.032	0.022	**0.036**	**0.017**	**0.075**	**0.021**	0.16	0.88	0.15
Job insecurity	0.018	0.015	0.023	0.012	0.017	0.014	0.98	0.77	0.73
Work life conflict	**0.043**	**0.015**	**0.026**	**0.012**	0.019	0.014	0.24	0.39	0.71
Vertical trust + organizational justice	**0.265**	**0.025**	**0.220**	**0.020**	**0.222**	**0.023**	0.21	0.17	0.96

Employed Canadians working in workplaces with more than five employees (*N* = 3919). Adjusted for age, sex, language, workplace size, province or territory of employment, industry of employment, level of education, shift schedule, management role, and multiple employment. Bold β coefficients and SE indicate a significant difference from the null at the 95% confidence level. Psychosocial exposures were adjusted to a 0 to 10 scale, with higher scores indicating a more negative exposure. The outcome was measured using a single question “How would you rate the workplace psychological health and safety climate in your workplace?” with response options being healthy/supportive, good, fair, neutral, not so good, poor, and toxic. The outcome was scored such that a workplace worse psychological health and safety climate was given a higher score on a scale of 1 to 7. Positive β coefficients indicate a higher level of the psychosocial exposure is associated with a worse psychological health and safety climate. Bold *P* values indicate a significant difference between compared β coefficients at the 95% confidence level. *N*_Masculine_ = 1011, model *R* ^2^_Masculine_ = 0.61; *N*_Intermediate_ = 1748, model *R* ^2^_Intermediate_ = 0.57; *N*_Feminine_ = 1160, and model *R* ^2^_Feminine_ = 0.61.

Abbreviation: COPSOQ, Copenhagen Psychosocial Questionnaire.

**Table 6:** Standardized ordinary least‐squared linear regression estimates for dimensions of the COPSOQ and perceived psychological health and safety in the workplace, stratified by age groups

	<30 y (*N* = 511)		30 to 50 y (*N* = 1747)		>50 y (*N* = 1661)		*P* values for difference		
COPSOQ dimensions	β Coefficient	SE	β Coefficient	SE	β Coefficient	SE	1 vs 3	1 vs 2	2 vs 3
Quantitative demands	−0.030	0.030	**0.044**	**0.016**	0.001	0.016	0.34	**0.03**	**0.05**
Work pace	0.031	0.026	−0.004	0.014	0.008	0.014	0.43	0.23	0.53
Emotional demands	**0.129**	**0.026**	**0.075**	**0.014**	**0.096**	**0.015**	0.27	0.07	0.32
Influence at work	0.011	0.024	**0.053**	**0.014**	**0.060**	**0.014**	0.08	0.13	0.71
Possibilities for development	−0.002	0.029	**−0.068**	**0.018**	−0.010	0.019	0.81	0.05	**0.03**
Meaning of work + commitment to the workplace	0.063	0.030	**0.084**	**0.018**	**0.054**	**0.018**	0.79	0.56	0.24
Predictability + rewards	0.072	0.040	**0.137**	**0.023**	**0.116**	**0.023**	0.34	0.15	0.51
Role clarity	−0.043	0.030	**−0.050**	**0.015**	−0.015	0.016	0.39	0.85	0.11
Role conflict	0.013	0.025	**0.030**	**0.015**	0.008	0.015	0.88	0.55	0.31
Quality of leadership + social support from supervisors	**0.089**	**0.034**	**0.065**	**0.017**	**0.079**	**0.016**	0.78	0.52	0.56
Social support from colleagues	0.007	0.028	0.004	0.014	−0.030	0.015	0.25	0.92	0.10
Social community at work	**0.084**	**0.030**	**0.054**	**0.017**	0.024	0.018	0.09	0.38	0.23
Job insecurity	0.000	0.023	**0.026**	**0.012**	0.021	0.012	0.41	0.33	0.80
Work life conflict	0.018	0.021	0.021	0.012	**0.040**	**0.012**	0.37	0.90	0.28
Vertical trust + organizational justice	**0.206**	**0.035**	**0.221**	**0.019**	**0.237**	**0.020**	0.44	0.70	0.56

Employed Canadians working in workplaces with more than five employees (*N* = 3919). Adjusted for sex, gender role, language, workplace size, province or territory of employment, industry of employment, level of education, shift schedule, management role, and multiple employment. Psychosocial exposures were adjusted to a 0 to 10 scale, with higher scores indicating a more negative exposure. The outcome was measured using a single question “How would you rate the workplace psychological health and safety climate in your workplace?” with response options being healthy/supportive, good, fair, neutral, not so good, poor, and toxic. The outcome was scored such that a worse workplace psychological health and safety climate was given a higher score on a scale of 1 to 7. Positive β coefficients indicate a higher level of the psychosocial exposure is associated with a worse psychological health and safety climate. Bold β coefficients and SE indicate a significant difference from the null at the 95% confidence level. Bold *P* values indicate a significant difference between compared β coefficients at the 95% confidence level. *N*_Age<30_ = 511, model *R* ^2^_Age<30_ = 0.53; *N*_30<Age<50_ = 1747, model *R* ^2^_Age<30_ = 0.58; *N*_Age>50_ = 1661, and model *R* ^2^_Age>50_ = 0.62.

Abbreviation: COPSOQ, Copenhagen Psychosocial Questionnaire.

## 4 DISCUSSION

We observed that there are two dimensions of the psychosocial work environment that are consistently important across all workers regardless of sex, gender role, or age. These were quality of leadership and social support from supervisors, and vertical trust and organizational justice. Along with predictability and rewards, and meaning of work and commitment to the workplace, these dimensions generally had the strongest associations with the global rating of workplace psychological health and safety across demographic subgroups. These findings suggest that organizational leadership, which is related to justice, trust, and the ability to resolve problems, plays an important role in determining much of workplace psychological health and safety. It is important to recognize the psychosocial exposures that have an influence on all working Canadians, so that approaches to improving psychosocial health can be targeted towards those dimensions of the workplace. Identifying a few key dimensions may be beneficial for workplaces to develop greater efficacy for change, especially for smaller workplaces with limited resources.^24^ Few differences were observed across sex, gendered labor market roles, and age groups; only six differences were found among all subgroup comparisons. Given that 105 differences were examined, we would expect six differences to be present based on chance alone. As such, it is not clear whether these differences observed are spurious relationships or not.

Estimates between specific psychosocial work exposures and the global rating of workplace psychological health and safety were attenuated to a large extent in models with all psychosocial work exposures, compared with models with only single psychosocial work exposures (Table 3). This observation demonstrates the complex relationships between dimensions of the psychosocial work environment, and challenges in isolating the effects associated with single psychosocial dimensions. To isolate the total effects of each psychosocial exposure would require greater specificity about the relationships between workplace dimensions. This is because upstream (distal) dimensions should be included in models to examine the effects of more proximal dimensions, while proximal dimensions should not be included in models for more distal dimensions.^25^ For example, in recent studies of the COPSOQ, leadership resources have been positioned as distal factors the lead to differences in job demands and positive work attitudes, with subsequent impact on workability.^26^ However, more work is required to conceptualize how each of the dimensions in the COPSOQ relate to each other. In our adjusted model, we also observed that possibilities for development and social support from colleagues had inverse and statistically significant associations with the global rating of psychological health and safety at work, indicating that more negative exposures were associated with better psychological health and safety. These relationships may have been produced through overadjustment, since the inverse associations became nonsignificant if the meaning of work and commitment to the workplace were removed from the model. One of the challenges moving forward is identifying specific psychosocial factors that are important to different worker subgroups. Here, we have examined the differences across sex, gender, and age groups. However, there may also be observable differences across labor market groups based on immigrant status or duration of employment, for example.

As with any cross‐sectional survey design, this study has some inherent limitations. Since we are capturing a single point in time, it is not possible to draw any conclusions about causal relationships between the psychosocial dimensions of the workplace and the psychological health and safety climate. Cross‐sectional studies may also suffer from selection bias or information bias; however, the use of a representative panel of respondents in this study alleviates some of those concerns. A benefit of this cross‐sectional survey is that it allows for capture of a large sample of complete and complex data, and permits a detailed analysis of the working population. Another advantage of this study is that it includes novel measures in this population for which there are few missing observations, within a large and demographically diverse sample of workers. This allows broad conclusions to be made about the psychosocial exposures that are important to working‐age respondent's psychological health and safety. This is the only data set of this size and quality that captures this information; however, there is an underrepresentation of certain groups of workers, such as those under 30 and non‐English speakers, making it difficult to determine if the results of these analyses are applicable to those groups. Another limitation to this study is the lack of information on the nonrespondents and the low response rate (10%), which may be due to the length of the survey, or other factors that inhibit participation. Despite the low response rate, we observed good variance across the psychosocial exposure measures as well as the outcome measure (Table 2), allowing for examination of the relationships between these psychosocial dimensions and our psychological health and safety outcome.^27^ A final limitation is the single‐item used to assess the psychological health and safety climate. As outlined previously, this measure was created in the absence of an existing global measure of psychological health and safety at the worker level. While global assessments have proved an efficient way to assess aspects of health and job satisfaction and job stress in previous studies, the cognitive process used by respondents to answer a single‐item, or the ability of a single‐item to assess psychological health and safety has not been established.^28^, ^29^, ^30^, ^31^ That said, the measure used in this study did display excellent test‐retest reliability, with a very low percentages of missing responses (<1% of the sample did not answer this question), indicating the measure was consistently assessed by respondents, with almost all respondents in our sample feeling they were able to use one of the available seven categories to describe the psychological health and safety of their work environment.

The dimensions with the strongest associations with the global rating of the psychological health and safety at work were quality of leadership, social support from supervisors, vertical trust, organizational justice, predictability, rewards, meaning of work, and commitment to the workplace. Future work in this area should identify feasible and acceptable approaches to improve these dimensions of work, which could potentially lead to improvements in workplace psychological health and safety. It should be noted that interventions for improving the psychosocial work environment are likely dependent upon specific characteristics in the workplace, with successful interventions on the psychosocial environment require participatory, and likely multimodal, approaches.^32^, ^33^, ^34^ Systematic reviews in this area suggest that organization‐level participation interventions may have greater benefits for employee health, compared with task restructuring interventions, with another study suggesting multifaceted approaches to improving the psychosocial workplace environment are better than interventions targeting a single aspect of the workplace.^35^, ^36^, ^37^ Care should be taken when considering potential interventions related to psychosocial health; the use of a standardized questionnaire, such as the COPSOQ, might give a better indication of the present state of the workplace, and identify core dimensions to be improved.

Future work may also include the addition of multiple items to better measure the psychological health and safety in the workplace, and validation of those questions, as this outcome measure is not part of the standardized COPSOQ questionnaire. In addition, it would be valuable to explore how changes to each psychosocial dimension may lead to concurrent changes in psychological health and safety.

It is important to recognize the aspects of work environments that have an influence on the psychological health and safety of workers, so that appropriate dimensions of the work environment may be identified and remediated. It is also important to note that the effects of several important psychosocial exposures may differ between worker subpopulations based on sex, gender, and age. The results of our study suggest there are several dimensions of the work environment that are important to all workers’ psychological health and safety, and general approaches for improvement should consider these dimensions when designing and implementing workplace interventions.

## CONFLICTS OF INTEREST

The authors declare that there are no conflicts of interest.

## DISCLOSURE BY AJIM EDITOR OF RECORD

None.

## AUTHOR CONTRIBUTIONS

PS and JO conceived and designed this project. Acquisition, analysis, and interpretation of the data were conducted by all authors. AR and PS drafted the manuscript, with revisions and final approval from all authors. All authors agree to be accountable for all aspects of the work in ensuring that questions related to the accuracy or integrity of any part of the work are appropriately investigated and resolved.

## Supporting information

Supporting information
