# Supplementary material for: Dissecting the effect of workplace exposures on workers’ rating of psychological health and safety
Source: Am J Ind Med. 2019 Mar 27;62(5):412–21. doi: 10.1002/ajim.22964 (PMC6849279; doi:10.1002/ajim.22964)
Supplement: Supplementary file 1 — Supporting information [file AJIM-62-412-s001.doc]

Supplementary Material

A. COPSOQ Questionnaire

Primary Outcome

How would you rate the **psychological health & safety climate** in your workplace?

healthy/ supportive 1

good 2

fair 3

neutral 4

not so good 5

poor 6

toxic 7

The following questions are about your psychosocial work environment. Please choose the answer that fits best to each of the questions.

Quantitative Demands

Do you get behind with your work?

Always 1

Often 2

Sometimes 3

Seldom 4

Never/ hardly ever 5

Do you have enough time for your work tasks?

Always 1

Often 2

Sometimes 3

Seldom 4

Never/ hardly ever 5

How often do you not have time to complete all your work tasks?

Always 1

Often 2

Sometimes 3

Seldom 4

Never/ hardly ever 5

Work Pace

Do you have to work very fast?

Always 1

Often 2

Sometimes 3

Seldom 4

Never/ hardly ever 5

Do you work at a high pace throughout the day?

Always 1

Often 2

Sometimes 3

Seldom 4

Never/ hardly ever 5

Emotional Deamands

Does your work put you in emotionally disturbing situations?

Always 1

Often 2

Sometimes 3

Seldom 4

Never/ hardly ever 5

Do you have to deal with other people's personal problems as part of your work?

Always 1

Often 2

Sometimes 3

Seldom 4

Never/ hardly ever 5

Is your work emotionally demanding?

To a very large extent 1

To a large extent 2

Somewhat 3

To a small extent 4

To a very small extent 5

**Influence at work**

Do you have a large degree of influence on the decisions concerning your work?

Always 1

Often 2

Sometimes 3

Seldom 4

Never/ hardly ever 5

Can you influence the amount of work assigned to you?

Always 1

Often 2

Sometimes 3

Seldom 4

Never/ hardly ever 5

Possibilities for Development

Do you have the possibility of learning new things through your work?

To a very large extent 1

To a large extent 2

Somewhat 3

To a small extent 4

To a very small extent 5

Can you use your skills or expertise in your work?

To a very large extent 1

To a large extent 2

Somewhat 3

To a small extent 4

To a very small extent 5

Does your work require you to take the initiative?

To a very large extent 1

To a large extent 2

Somewhat 3

To a small extent 4

To a very small extent 5

Meaning of work

Is your work meaningful?

To a very large extent 1

To a large extent 2

Somewhat 3

To a small extent 4

To a very small extent 5

Do you feel that the work you do is important?

To a very large extent 1

To a large extent 2

Somewhat 3

To a small extent 4

To a very small extent 5

**Commitment to the workplace**

Do you feel that your place of work is of great importance to you?

To a very large extent 1

To a large extent 2

Somewhat 3

To a small extent 4

To a very small extent 5

Would you recommend other people to apply for a position at your workplace?

To a very large extent 1

To a large extent 2

Somewhat 3

To a small extent 4

To a very small extent 5

**Predictability**

At your place of work, are you informed well in advance concerning for example important decisions, changes, or plans for the future?

To a very large extent 1

To a large extent 2

Somewhat 3

To a small extent 4

To a very small extent 5

Do you receive all the information you need in order to do your work well?

To a very large extent 1

To a large extent 2

Somewhat 3

To a small extent 4

To a very small extent 5

**Rewards**

Is your work recognised and appreciated by the management?

To a very large extent 1

To a large extent 2

Somewhat 3

To a small extent 4

To a very small extent 5

Are you treated fairly at your workplace?

To a very large extent 1

To a large extent 2

Somewhat 3

To a small extent 4

To a very small extent 5

**Role Clarity**

Does your work have clear objectives?

To a very large extent 1

To a large extent 2

Somewhat 3

To a small extent 4

To a very small extent 5

Do you know exactly what is expected of you at work?

To a very large extent 1

To a large extent 2

Somewhat 3

To a small extent 4

To a very small extent 5

**Role Conflicts**

Are contradictory demands placed on you at work?

To a very large extent 1

To a large extent 2

Somewhat 3

To a small extent 4

To a very small extent 5

Do you sometimes have to do things which ought to have been done in a different way?

To a very large extent 1

To a large extent 2

Somewhat 3

To a small extent 4

To a very small extent 5

Do you sometimes have to do things which seem to be unnecessary?

To a very large extent 1

To a large extent 2

Somewhat 3

To a small extent 4

To a very small extent 5

**Quality of Leadership**

To what extent would you say that your immediate superior gives high priority to job satisfaction?

To a very large extent 1

To a large extent 2

Somewhat 3

To a small extent 4

To a very small extent 5

To what extent would you say that your immediate superior is good at work planning?

To a very large extent 1

To a large extent 2

Somewhat 3

To a small extent 4

To a very small extent 5

To what extent would you say that your immediate superior is good at solving conflicts?

To a very large extent 1

To a large extent 2

Somewhat 3

To a small extent 4

To a very small extent 5

**Social Support from Supervisors**

How often would your immediate superior be willing to listen to your problems at work, if needed?

Always 1

Often 2

Sometimes 3

Seldom 4

Never/ hardly ever 5

How often would you get help and support from your immediate superior, if needed?

Always 1

Often 2

Sometimes 3

Seldom 4

Never/ hardly ever 5

**Social Support from Colleagues**

How often could you get help and support from your colleagues, if needed?

Always 1

Often 2

Sometimes 3

Seldom 4

Never/ hardly ever 5

**Social Community at work**

Is there a good atmosphere between you and your colleagues?

Always 1

Often 2

Sometimes 3

Seldom 4

Never/ hardly ever 5

**Job Insecurity**

Are you worried about becoming unemployed?

To a very large extent 1

To a large extent 2

Somewhat 3

To a small extent 4

To a very small extent 5

Are you worried about it being difficult for you to find another job if you became unemployed?

To a very large extent 1

To a large extent 2

Somewhat 3

To a small extent 4

To a very small extent 5

Are you worried about being transferred to another job against your will?

To a very large extent 1

To a large extent 2

Somewhat 3

To a small extent 4

To a very small extent 5

**Satisfaction with work**

Regarding your work in general, how pleased are you with your job as a whole, everything taken into consideration?

Very satisfied 1

satisfied 2

Neither/ nor 3

Unsatisfied 4

Very unsatisfied 5

**Work-Life Conflict**

The next three questions are about the way your work affects your private life and family life.

Do you feel that your work drains so much of your energy that it has a negative effect on your private life?

Yes, certainly 1

Yes, to a certain degree 2

Yes, but only very little 3

No, not at all 4

Do you feel that your work takes so much of your time that it has a negative effect on your private life?

Yes, certainly 1

Yes, to a certain degree 2

Yes, but only very little 3

No, not at all 4

Are there times when you need to be at work and at home at the same time?

Yes, certainly 1

Yes, to a certain degree 2

Yes, but only very little 3

No, not at all 4

**Vertical Trust**

The next four questions are not about your own job but about *the whole company* you work at.

Can the employees trust the information that comes from the management?

To a very large extent 1

To a large extent 2

Somewhat 3

To a small extent 4

To a very small extent 5

Does the management trust the employees to do their work well?

To a very large extent 1

To a large extent 2

Somewhat 3

To a small extent 4

To a very small extent 5

**Organisational Justice**

Are conflicts resolved in a fair way?

To a very large extent 1

To a large extent 2

Somewhat 3

To a small extent 4

To a very small extent 5

Is the work distributed fairly?

To a very large extent 1

To a large extent 2

Somewhat 3

To a small extent 4

To a very small extent 5

**B. Demographic comparison of the study participants and the Canadian Labour Force**

|  | **Study Participants** | **Canadian Labour Force*** |
| --- | --- | --- |
| **Age Group** |  |  |
| Less than 30 years | 13% | 26% |
| 30 to 49 years | 45% | 44% |
| 50 or older | 42% | 30% |
| **Sex** |  |  |
| Male | 48% | 50% |
| Female | 52% | 50% |
| **Highest Education** |  |  |
| Less than secondary | 3% | 9% |
| Secondary graduation | 12% | 25% |
| Post-secondary below bachelors | 35% | 36% |
| Bachelors degree or higher | 51% | 30% |
| **Full-time Work** |  |  |
| Yes | 81% | 81% |
| No | 19% | 19% |
| **Multiple Job Holder** |  |  |
| Yes | 88% | 95% |
| No | 12% | 5% |
| **Workplace Size** |  |  |
| 5 to 20 employees | 21% | 32% |
| 21 to 99 employees | 31% | 33% |
| 100 to 499 employees | 27% | 21% |
| 500 or more employees | 21% | 14% |
| **Industry of Main Employment** |  |  |
| Manufacturing | 7% | 11% |
| Trades, transport and warehousing | 6% | 9% |
| Other goods industries | 7% | 9% |
| Educational services | 15% | 8% |
| Health care and social assistance | 14% | 14% |
| Retail trade and accomodation | 9% | 20% |
| Other service industries | 42% | 30% |

* Estimates from Statistics Canada's Labour Force Survey for February and March 2016. Estimates account for all employed workers, including those in workplaces with less than 5 employees.
